# Organic Liquid Fertilizer Coupled With Single Application of Chemical Fertilization Improves Growth, Biomass, and Yield Components of Cotton Under Mulch Drip Irrigation

**DOI:** 10.3389/fpls.2021.763525

**Published:** 2022-01-20

**Authors:** Xiao-juan Shi, Xian-zhe Hao, Nan-nan Li, Jun-hong Li, Feng Shi, Huan-yong Han, Yu Tian, Yun Chen, Jun Wang, Hong-hai Luo

**Affiliations:** ^1^The Key Laboratory of Oasis Eco-Agriculture, Xinjiang Production and Construction Group, Shihezi University, Shihezi, China; ^2^Xinjiang Academy Agricultural and Reclamation Science, Shihezi, China

**Keywords:** organic liquid fertilizer, cotton, agronomic trait, biomass accumulation, yield

## Abstract

Excessive fertilization, low nutrient utilization rate, and continuous deterioration of cotton field environment have adversely affected the sustainable development of cotton in Xinjiang province of China. To overcome these issues, we hypothesized that an appropriate combination of liquid organic fertilizer and chemical fertilizer (CF) would effectively reduce the input of CF without sacrificing the quality and yield of cotton. A 2-year field experiment explores the effects of three fertilization treatments on the growth, biomass accumulation, and yield of cotton. The three fertilization treatments, namely, no application of fertilizer (CK), the single application of CF, and the combined application of organic liquid fertilizer and CF (F0.6–F1.4), were set up in five ratios. Compared with CF treatment, the combined application of organic liquid fertilizer and CF treatments (F0.6–F1.2) speeded the growth period of cotton by 2–7 days with increased plant height, stem diameter, functional leaf width, and more number of branches, with 9.7–23.5 and 8.4–28.5% higher total plant biomass (TPB) and reproductive organs biomass (ROB), respectively. Compared with CF treatment, the rapid growth duration and maximum accumulation rate of reproductive organs were the highest in F0.8 treatment, with an average increase of 4.6 days and 20.3%. Increment in biomass accumulation contributed to an average increase of 21.8 and 18.9% in cotton boll number and yield, respectively, under F0.8 treatment. Principal component analysis shows that the total biomass, ROB, and total bolls per unit area were positively correlated with the yield, while stem diameter and vegetative organ biomass are negatively correlated with the yield. In conclusion, under film mulching with drip irrigation, organic liquid fertilizer combined with CF reduced by 20% (F0.8 treatment: N, P_2_O_5_, and K_2_O were 182, 104, and 76 kg hm^–2^, respectively) can sustain the normal growth, promote the accumulation rate of ROB, and lead to efficient cotton production.

## Introduction

China had a 25.6% of the cotton production worldwide in 2020, and its seeded area was 10.1% of the total world (Arshad et al., 2021). Xinjiang, being a high-quality cotton production area in China, had 25,019 km^2^ of cotton planting area, accounting for 78.9% of the total in the country. The total cotton output reached 5.161 million tons, accounting for 87.3% of the national total output and 22.3% of the global total cotton output (National Bureau of Statistics, 2020). Xinjiang has become an important region that affects the world cotton production pattern (Lou et al., 2021). However, with the rising labor cost, agricultural materials, and logistics, the relationship of the cost and profit of cotton production has gradually entered a stage of “high input and low output” (Li et al., 2016). The pursuit of high yield is often accompanied by the increasing input of chemical fertilizer (CF) (Dong et al., 2010). However, the problems of soil fertility degradation, greenhouse gas emission, imbalance of soil structure, and soil-borne diseases are becoming serious year by year (Li et al., 2017; Nan et al., 2019; Massey and Gedikoglu, 2021). Therefore, to ensure the efficient and sustainable development of cotton in Xinjiang, exploring a reasonable fertilization model has become one of the key challenges for cotton researchers.

Appropriate application of organic fertilizer and CF is a crucial measure to improve the fertilizer utilization rate and solve the problem of unreasonable use of CF (Han et al., 2021). It has been found that the yield of seed cotton with organic fertilizer combined with CF increases significantly (Tian et al., 2012). Also, the application of organic fertilizer could improve the composition of soil carbon pool and the transformation process and thus raise the soil quality (Zhang P. P. et al., 2019). However, the continuous increase of the replacement amount of organic fertilizer will also lead to the reduction in crop yield, which is attributed to the reduction of the accumulation and operation of dry matter and NPK nutrients during the growth period (Pei et al., 2020). Therefore, there is no definite standard for the amounts of organic fertilizer for different crops, and the standardized use of organic fertilizer is the premise of improving the quality and production efficiency of crops.

Film mulching with drip irrigation improves the utilization efficiency of water and fertilizer and is widely used in arid areas (Chen et al., 2010). It is easy to form a smaller root-to-shoot ratio through drip irrigation under mulch, which is beneficial to the distribution of photosynthetic products to reproductive organs and improves the yield of seed cotton (Wang et al., 2020). However, the traditional organic fertilizer application method has low utilization rate and poor solubility, which increases the cost and is not conducive to simplifying cotton planting (Tao et al., 2014). Our previous research found that organic liquid fertilizer can regulate cotton plant type, promote cotton boll maturity, and increase cotton yield (Ma et al., 2020). However, few reports are available on the mechanism of increased cotton production and dry matter accumulation characteristics of the combined application of organic liquid fertilizer and CF under drip irrigation with plastic film mulching. Furthermore, the amount of the combined application of organic liquid fertilizer and CF has not yet been explored.

Therefore, the purpose of this study was to explore the effects of the combined application of organic liquid fertilizer and CF on cotton growth and agronomic traits and to determine the relation of cotton growth, agronomic traits, and biomass accumulation with yield using the principal component analysis. This study also explores and finds out the best application amount of the combined application of organic liquid fertilizer and CF suitable for high and stable yield of cotton.

## Materials and Methods

### Experimental Site and Cultivar

**FIGURE 1:**
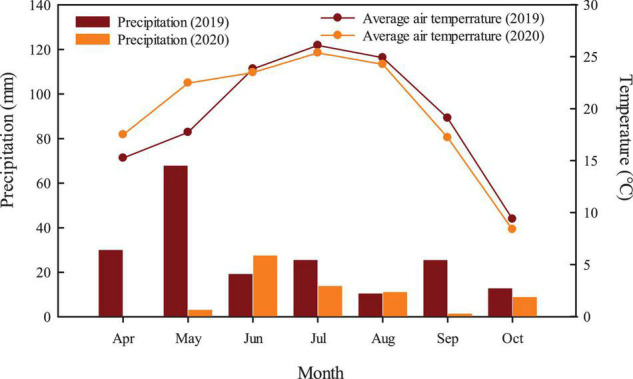
Month average air temperature and rainfall in growth period of cotton in 2019 and 2020.

The experiment was conducted at the Crop Efficient Water Use Observatory (45°38′N, 86°09′E) of the Ministry of Agriculture of Xinjiang Academy of Agricultural Sciences from 2019 to 2020. It was largely sandy loam, and the former crop of the experimental area was corn. The basic physical and chemical properties of the 0–20 cm topsoil are as follows: organic matter 15.00 g kg^–1^, alkali-hydrolyzable nitrogen 42.20 mg kg^–1^, rapid available phosphorus 19.81 mg kg^–1^, and rapid available potassium 274.28 mg kg^–1^. The tested cotton was Xinluzao 74 (*Gossypium hirsutum* L.), which had a growth period of 124 days and was provided by Shihezi Academy of Agricultural Sciences. The average temperature and precipitation during cotton growth period are shown in Figure 1.

### Experimental Design and Management

In this study, three fertilization treatments were targeted, i.e., specific application rates of nitrogen, phosphorus, and potassium in each treatment (Table 1); no application of fertilizer (CK), the single application of CF, and the combined application of organic liquid fertilizer and CF were set up in five ratios. For CF treatment, N, P_2_O_5_, and K_2_O were 228, 131, and 95 kg hm^–2^, respectively. The F0.6–F1.4 treatments only adjusted the application of N, P_2_O_5_, and K_2_O, with 137, 78, and 57 kg hm^–2^ for the treatment F0.6 (organic liquid fertilizer combined with CF reduced by 40%); 182, 104, and 76 kg hm^–2^ for the treatment F0.8 (organic liquid fertilizer combined with CF reduced by 20%); 228, 131, and 95 kg hm^–2^ for the treatment F1.0 (organic liquid fertilizer combined with CF); 273, 157, and 114 kg hm^–2^ for the treatment F1.2 (organic liquid fertilizer combined with CF increased by 20%); and 319, 183, and 133 kg hm^–2^ for the treatment F1.4 (organic liquid fertilizer combined with CF increased by 40%), respectively. The experiment was performed in a randomized complete design with three replications. Each plot was 10 m long and 6.9 m wide.

**TABLE 1:** Fertilization schemes for different treatments (kg hm^–2^).

Treatment	Squaring fertilizer			Flowering and boll setting fertilizer			Total nutrient content		
	N	P_2_O_5_	K_2_O	N	P_2_O_5_	K_2_O	N	P_2_O_5_	K_2_O
CK	0	0	0	0	0	0	0	0	0
CF	83.25	45.00	21.75	144.38	85.50	73.50	227.63	130.50	95.25
F1.0	83.25	45.00	21.75	144.38	85.50	73.50	227.63	130.50	95.25
F0.6	49.95	27.00	13.05	86.63	51.30	44.10	136.58	78.30	57.15
F0.8	66.60	36.00	17.40	115.50	68.40	58.80	182.10	104.40	76.20
F1.2	99.90	54.00	26.10	173.25	102.60	88.20	273.15	156.60	114.30
F1.4	116.55	63.00	30.45	202.13	119.70	102.90	318.68	182.70	133.35

**FIGURE 2:**
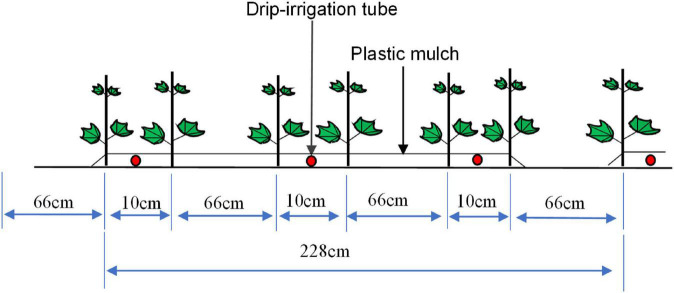
Schematic diagram of the planting mode (one mulch and six rows of cotton plants).

Chemical fertilizer treatment included urea (containing N 46.0%), monoammonium phosphate (ammonium dihydrogen phosphate containing N 12.0% and P_2_O_5_ 61.0%), and potassium sulfate (containing K_2_O 50.0%). Organic liquid fertilizer was developed by our research group, with yeast waste liquid as carrier, adding trace elements, functional microbial flora. The effective components were soluble organic matters (humic acid ≥ 30 g ^–1^, amino acid ≥ 10 g L^–1^), trace elements (manganese, zinc, boron ≥ 1 g L^–1^), microbial flora (*Bacillus subtilis* ≥ 2 × 10^8^ g^–1^), and pH 4.5–6.5. Cotton was grown by drip irrigation combined with the plastic film mulching technique. The film was 2.28 m wide, with six rows in each film sheet (row spacings of 10, 66, 10, 66, 10, and 66 cm; see Figure 2), sown on April 18, 2019 and April 13, 2020. The total irrigation amount was 4350 m^3^ hm^–2^, all treated fertilizers were applied topdressing under drip irrigation, and other management measures were the same as those of high-yield cotton fields, except fertilizer factors.

### Data Collection

#### Growth Process and Agronomic Traits

Ten cotton plants with uniform growth were continuously fixed in each treatment, and the actual date of each period [bud emergence, flowering, full flowering, full boll, and boll opening (BO)] was recorded every 7 days. The plant height, number of fruit branches, width of inverted four leaves, stem diameter, and number of buds, flowers, and bolls of cotton were investigated in each growth period.

#### Yield and Its Components

At the harvest period (September 22, 2019 and September 26, 2020), three representative sampling points (2.93 m × 2.28 m) were selected for each treatment, and the number of plants per unit area and bolls per plant in each sampling point were investigated. Meanwhile, 50 cotton bolls were picked from the top, middle, and bottom of each plot (according to the plant height, above one-third of the height was the upper branch, two-thirds was the middle branch, and the rest was the lower branch), which were put into paper bags separately for indoor determination of lint percentage and single boll weight.

#### Biomass Accumulation and Distribution

Six plants were sampled at budding, full flowering, full boll, later full boll (LFB), and BO periods of cotton growth. Plants were divided into different organs, such as roots (below cotyledon knot), stems, leaves, and buds. These samples were enveloped and placed in oven at 105°C for 0.5 h. Then, the temperature was lowered to 80°C, and the plant samples were dried to constant weight and weighed to record dry weight.

A logistic equation was used to describe the process of biomass accumulation (Yang et al., 2011).


(1) $$\text{Y}=\frac{K}{1+ae^{bt}}$$

where *Y* (g) represents the biomass at the actual time *t*, *K* (g) denotes the maximum biomass, *t* (days) is the number of days after emergence (DAE), and *a* and *b* are constants.

From Eq. 1, it follows that


(2) $$t_{0}=\frac{ln^{a}}{b}$$

(3) $$t_{1}=\frac{1}{b}ln^{\left(\frac{2+\sqrt{3}}{a}\right)}$$

(4) $$t_{2}=\frac{1}{b}ln^{\left(\frac{2-\sqrt{3}}{a}\right)}$$

(5) $$V_{m}=-\frac{bK}{4}$$

(6) $$V_{t}=\frac{Y_{2}-Y_{1}}{t_{2}-t_{1}}$$

When *t* = *t*_0_, *V*_*m*_ (g day^–1^) is the maximum biomass accumulation rate in the rapid accumulation period, *t*_0_ (days) is rapid biomass accumulation period, *t*_1_ (days) is the start point of biomass rapid accumulation period, *t*_2_ (days) is the end point of biomass rapid accumulation period, *Y*_1_ (g) and *Y*_2_ (g) are the biomass accumulation on the *t*_1_-th and *t*_2_-th days, respectively, and *V*_*t*_ is the average biomass accumulation rate from *t*_1_ to *t*_2_.

### Statistical Analysis

Microsoft Office 2010 was used for data processing. Figures were plotted using Sigmaplot 14.0 software. R 4.0.4 was used to analyze the principal component analysis, SPSS 19.0 for data variance analysis and regression analysis, and Duncan’s method for multiple comparison. The significant difference level was 0.05 (*P* < 0.05).

## Results and Analysis

### Growth Period

Cotton plant transitioned to squaring stage, flowering stage, and BO stage at 34–35, 55–62, and 114–127 days, respectively (Table 2). The growth period of cotton plant increased with the increase of organic liquid fertilizer and CF in the following trend: F1.4 > F1.2 > F1.0 > F0.8 > F0.6. Compared with CF treatment, the growth period of F1.0 treatment was 4 days earlier, mainly because the flowering and BO of the F1.0 treatment were 4 days earlier. However, compared with CF treatment, the growth period of F0.6 and F0.8 treatments was 5–8 more days earlier; squaring period, flowering, and boll setting period were shortened by 6–8 days; and the flowering and boll setting period of F1.2 and F1.4 treatments increased by 3–4 days.

**TABLE 2:** Effect of different fertilization schedules on the cotton growing stages and periods (2020).

Treatment	Growing stage (month–day)				Growing period (day)			
	Emergence	Squaring	Bloom	Opening	Seeding	Squaring	Flowering and boll setting	Total
CF	4–28	6–2	6–28	9–1	35	26	65	126
F0.6	4–28	6–1	6–22	8–20	34	21	59	114
F0.8	4–28	6–2	6–25	8–23	35	23	59	117
F1.0	4–28	6–1	6–27	8–28	34	26	62	122
F1.2	4–28	6–1	6–26	8–30	34	25	65	124
F1.4	4–28	6–1	6–28	9–2	34	27	66	127

### Agronomic Traits

**FIGURE 3:**
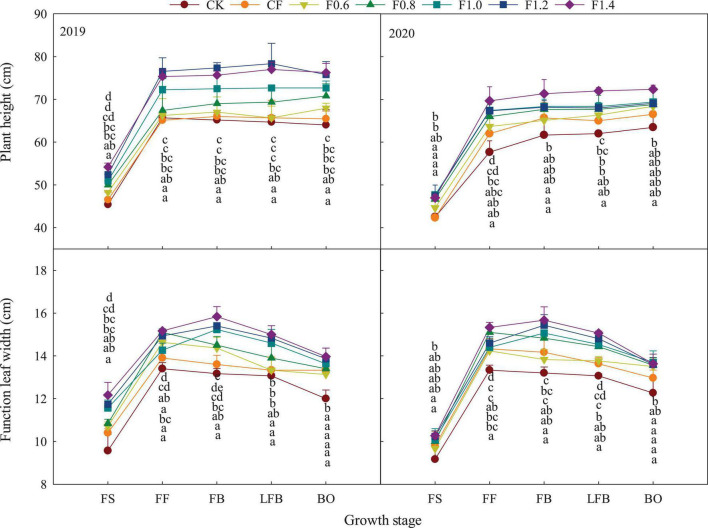
The changes of cotton plant height and functional leaf width in different fertilization treatments. FS, full squaring stage; FF, full flowering stage; FB, full boll stage; LFB, later full boll stage; BO, boll opening stage. Different letters above the adjacent seven columns indicate statistical significance at the *P* = 0.05 level.

The height of cotton plant increases rapidly before the full flowering period and sustained later in the season (Figure 3). Plant height among different treatments increases in the following order: F1.4 > F1.2 > F1.0 > F0.8 > F0.6 > CF. The functional leaf width increases first and then decreases as the plant transitioned from one stage to another stage. The functional leaf width was higher at peak at full flowering stage under CF treatment, while the combined application of organic liquid fertilizer and CF treatments had higher at peak at full boll stage. Compared with CF treatment, combined application of organic liquid fertilizer and the increased CF increased the functional leaf width at different stages, except for F0.6 treatment. Compared with F1.0 treatment, the leaf width of F0.8 treatment was significantly higher at full flowering stage.

**FIGURE 4:**
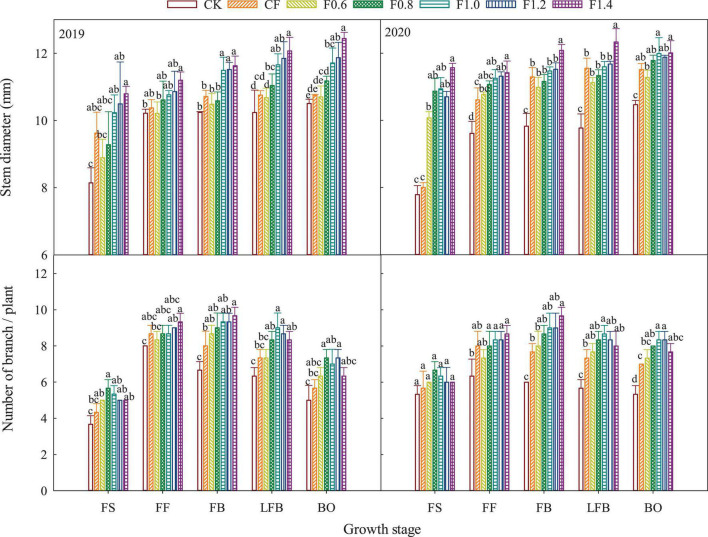
The changes of cotton stem diameter and cotton branches in different fertilization treatments. FS, full squaring stage; FF, full flowering stage; FB, full boll stage; LFB, later full boll stage; BO, boll opening stage. Different letters above the adjacent seven columns indicate statistical significance at the *P* = 0.05 level.

The stem diameter of cotton plant was higher at BO period (Figure 4). Before full flowering stage, the stem diameter of fertilization treatments was significantly higher than that of CK, and there was no significant difference among fertilization treatments. After full flowering, the stem diameter of F1.0, F1.2, and F1.4 treatments was significantly higher than that of CK, CF, and F0.6 treatments.

The number of branches of cotton plant first increased and then decreased with the growth period. Compared with CK treatment, the number of branches in different treatments increased significantly, and there was no obvious difference between F0.6 and CF treatments in the whole growth period. The number of branches in CK and CF treatments reached the peak at full flowering stage, while the combined application of organic liquid fertilizer and CF treatments had higher number at full boll stage. Compared with LFB stage, the number of effective branches of each treatment decreased at BO stage, among which the decreases of F0.8 and F1.2 treatments were small, 8.00 and 7.69%, respectively, and F1.4 treatment was the highest with 14.08%.

### Biomass Accumulation

**FIGURE 5:**
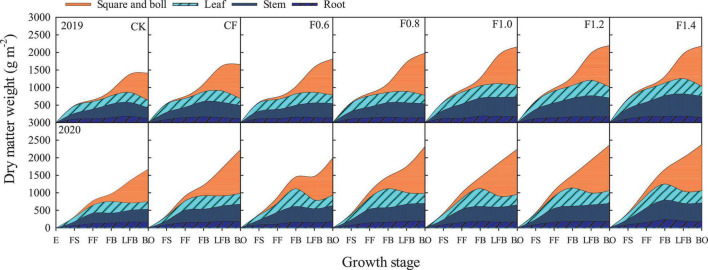
The change in productive organ, leaf, and stem, and root dry mass (g m^–2^). E, emergence; FS, full squaring stage; FF, full flowering stage; FB, full boll stage; LFB, later full boll stage; BO, boll opening stage.

The biomass accumulation of cotton plant was significantly influenced by different fertilization treatments (Figure 5). Biomass accumulation was increased when the plant grew. Compared with CF treatment, the total plant biomass (TPB) of F0.8–F1.4 treatments was increased by 9.7–23.5% and the vegetative organ biomass (VOB) of F0.8–F1.4 treatments was increased by 8.4–28.5%, but root biomass of F1.2 and F1.4 treatments were increased by 24.1 and 12%, respectively. The reproductive organ biomass (ROB) in different treatments had no significant effect at full flowering stage. Compared with CK treatment, the average increase of ROB in fertilization treatment was 36.9%. Compared with CF treatment, the ROB of F0.8 and F0.6 treatments were increased by 20.1 and 7.0%, respectively.

### Characteristics of Biomass Accumulation

Regression analysis (Table 3) shows that the accumulation of cotton biomass types in each treatment accords with logistic function. ROB was better than VOB since reproductive growth was more vigorous and the vegetative growth was inhibited after full flowering. Compared with CK treatment, the time (*t*_1_) periods when TPB and VOB entered the rapid accumulation period of cotton under fertilization treatment were 2.6 and 2 days shorter, respectively, while ROB was 0.8 days longer. The fast accumulation termination time (*t*_2_) shows that TPB and ROB in CF treatment took 1.2 more days, and VOB was shortened by 1.9 days. Under the combined application of organic liquid fertilizer and CF treatments, the termination time of TPB and ROB was delayed by 3.4 days, and VOB was greater by 0.1 day. Compared with CK treatment, the maximum and average accumulation speed (*V*_*m*_ and *V*_*t*_) of TPB, VOB, and ROB in CF treatment increased by 31.8 and 31.3, 20.2 and 20.2, and 29.2 and 29.2%, respectively, in the rapid accumulation period (*T*). *V*_*m*_ and *V*_*t*_ in the combined application of organic liquid fertilizer and CF treatments increased by 46.2 and 45.7, 49.1 and 49.3, and 39.7 and 39.9%, respectively. The fast accumulation duration of biomass in different treatments was F0.6 > F0.8 > F1.4 > F1.2 > F1.0 > CF for TPB, F0.6 > CF > F1.0 > F1.2 > F1.4 > F0.8 for VOB, and F0.8 > F0.6 > F1.2 > CF > F1.4 > F1.0 for ROB. Compared with CF treatment, *V*_*m*_ and *V*_*t*_ had the largest increase in F1.4 treatment, followed by F0.8 treatment; compared with CF treatment, *V*_*m*_ and *V*_*t*_ of TPB increased by 18.6 and 18.3, 15.9 and 15.7% on average; the average increase in VOB was 45.6 and 45.5, 32.6 and 32.7%; while ROB increased by 20.3 and 20.5%.

**TABLE 3:** Biomass accumulation equations of cotton in different organs and eigenvalues of cotton biomass accumulations under different fertilization treatments.

Year		Treatment	Regression equation	*R* ^2^	*t*_1_ (days)	*t*_2_ (days)	*T* (days)	*V*_*m*_ (g d^–1^)	*V*_*t*_ (g d^–1^)
	Total plant biomass	CK	*Y* = 1689.45/(1 + 19.49e^–0.0401t^)	0.9723	41.2	106.9	65.7	16.9	14.9
		CF	*Y* = 1938.21/(1 + 23.54e^–0.0434t^)	0.9785	42.4	103.1	60.7	21.0	18.4
		F0.6	*Y* = 2726.55/(1 + 17.66e^–0.0301t^)	0.9899	51.6	139.1	87.5	20.5	18.0
		F0.8	*Y* = 2775.74/(1 + 19.17e^–0.0326t^)	0.9909	50.2	130.9	80.8	22.6	19.8
		F1.0	*Y* = 2706.46/(1 + 22.75e^–0.0382t^)	0.9910	47.3	116.3	69.0	25.8	22.7
		F1.2	*Y* = 2727.09/(1 + 21.64e^–0.0382t^)	0.9902	46.0	115.0	69.0	26.0	22.8
		F1.4	*Y* = 2832.36/(1 + 16.46e^–0.0340t^)	0.9884	43.6	121.1	77.5	24.1	21.1
2019	Vegetative organs biomass	CK	*Y* = 756.58/(1 + 29.27e^–0.0798t^)	0.6641	25.8	58.8	33.0	15.1	13.2
		CF	*Y* = 818.07/(1 + 55.54e^–0.0957t^)	0.6504	28.2	55.7	27.5	19.6	17.2
		F0.6	*Y* = 838.53/(1 + 9.32e^–0.0602t^)	0.8863	15.2	59.0	43.8	12.6	11.1
		F0.8	*Y* = 843.46/(1 + 133.88e^–0.1154t^)	0.8914	31.0	53.8	22.8	24.3	21.3
		F1.0	*Y* = 1097.60/(1 + 41.51e^–0.0799t^)	0.9896	30.1	63.1	33.0	21.9	19.2
		F1.2	*Y* = 1111.17/(1 + 54.48e^–0.0868t^)	0.9025	30.9	61.2	30.3	24.1	21.1
		F1.4	*Y* = 1150.42/(1 + 57.88e^–0.0906t^)	0.8674	30.3	59.3	29.1	26.1	22.8
	Reproductive organs biomass	CK	*Y* = 877.01/(1 + 9390.94e^–0.0932t^)	0.9996	84.0	112.3	28.3	20.4	17.9
		CF	*Y* = 1053.17/(1 + 13525.02e^–0.1005t^)	0.9997	81.5	107.8	26.2	26.5	23.2
		F0.6	*Y* = 1137.87/(1 + 11901.37e^–0.0971t^)	0.9989	83.1	110.2	27.1	27.6	24.2
		F0.8	*Y* = 1345.10/(1 + 8992.46e^–0.0938t^)	0.9998	83.0	111.1	28.1	32.0	28.1
		F1.0	*Y* = 1166.27/(1 + 31755.07e^–0.1098t^)	0.9987	82.4	106.4	24.0	31.5	27.7
		F1.2	*Y* = 1386.56/(1 + 4653.28e^–0.0851t^)	0.9999	83.8	114.7	31.0	29.5	25.9
		F1.4	*Y* = 1347.69/(1 + 11381.63e^–0.0924t^)	0.9994	86.8	115.3	28.5	31.1	27.3

	Total plant biomass	CK	*Y* = 1942.23/(1 + 58.01e^–0.0462*t*^)	0.9988	59.4	116.4	57.0	22.4	19.7
		CF	*Y* = 2640.75/(1 + 78.84e^–0.0474t^)	0.9996	64.4	119.9	55.6	31.2	27.4
		F0.6	*Y* = 2164.96/(1 + 60.06e^–0.0495t^)	0.9518	56.1	109.3	53.2	26.8	23.5
		F0.8	*Y* = 2617.73/(1 + 64.33e^–0.0479t^)	0.9930	49.2	94.7	45.5	37.9	33.2
		F1.0	*Y* = 2467.38/(1 + 88.65e^–0.0534t^)	0.9958	59.3	108.6	49.3	32.9	28.9
		F1.2	*Y* = 2604.06/(1 + 86.35e^–0.0529t^)	0.9972	59.4	109.2	49.8	34.4	30.2
		F1.4	*Y* = 2526.17/(1 + 124.84e^–0.0598t^)	0.9899	58.7	102.7	44.0	37.8	33.1
2020	Vegetative organs biomass	CK	*Y* = 750.21/(1 + 162.71e^–0.0906t^)	0.9810	41.7	70.7	29.1	17.0	14.9
		CF	*Y* = 975.70/(1 + 104.21e^–0.0769t^)	0.9893	43.3	77.5	34.3	18.8	16.4
		F0.6	*Y* = 932.13/(1 + 194.10e^–0.092t^)	0.7376	42.9	71.6	28.6	21.4	18.8
		F0.8	*Y* = 1032.37/(1 + 372.22e^–0.1029t^)	0.9335	44.7	70.3	25.6	26.6	23.3
		F1.0	*Y* = 981.96/(1 + 446.14e^–0.1089t^)	0.8748	43.9	68.1	24.2	26.7	23.4
		F1.2	*Y* = 1066.74/(1 + 282.70e^–0.0987t^)	0.9392	43.8	70.5	26.7	26.3	23.1
		F1.4	*Y* = 1107.19/(1 + 469.90e^–0.1076t^)	0.8904	44.9	69.4	24.5	29.8	26.1
	Reproductive organs biomass	CK	*Y* = 956.22/(1 + 57991.60e^–0.1112t^)	0.9810	86.8	110.5	23.7	26.6	23.3
		CF	*Y* = 1320.81/(1 + 32973.55e^–0.1037t^)	0.9971	87.6	113.0	25.4	34.2	30.0
		F0.6	*Y* = 1165.97/(1 + 3840.74e^–0.0824t^)	0.9996	84.2	116.1	32.0	24.0	21.1
		F0.8	*Y* = 1534.20/(1 + 4581.74e^–0.0805t^)	0.9999	88.4	121.1	32.7	41.0	36.0
		F1.0	*Y* = 1317.73/(1 + 174436.06e^–0.1245t^)	0.9998	86.4	107.5	21.2	30.9	27.1
		F1.2	*Y* = 1334.16/(1 + 87384.94e^–0.1174t^)	0.9974	85.7	108.1	22.4	39.2	34.3
		F1.4	*Y* = 1349.16/(1 + 61831.83e^–0.1148t^)	0.9994	84.6	107.6	22.9	38.7	34.0

### Cotton Yield and Its Components

Cotton yield and its components were substantially affected by different fertilization treatments (Table 4). The performance of seed cotton and lint yield between treatments was as follows: F0.8 > F1.0, F1.2, F0.6 > CF, F1.4. Compared with CF treatment, seed cotton was increased by 21.8, 15.0, and 13.7% for F0.8, F1.0, and F1.2 treatments, respectively. No significant differences were noted for lint percentage and single boll weight among different treatments. The number of bolls per plant and total number of bolls per unit area were increased in the following order: F0.8 > F1.0 > F1.2 > F0.6 > CF > F1.4 > CK. Compared with CF treatment, the number of bolls per plant and total number of bolls per unit area were increased by 22.4 and 18.9%, respectively, under F0.8 treatment.

**TABLE 4:** Cotton yield and its components under different fertilization treatments.

Year	Treatment	Boll no. per plant	Plant no. (10^4^ hm^–2^)	Boll weight (g)	Lint percentage (%)	Seed yield (kg hm^–2^)	Lint yield (kg hm^–2^)
	CK	5.48 ± 0.24*c*	114.6 ± 0.9*c*	4.21 ± 0.03*b*	40.7 ± 1.3*a*	4826 d	1966 d
	CF	5.76 ± 0.18*c*	123.5 ± 2.3*c*	4.69 ± 0.22*a*	40.7 ± 1.4*a*	5793 c	2359 c
	F0.6	6.36 ± 0.18*b*	132.8 ± 4.5*b*	4.52 ± 0.28*ab*	42.2 ± 1.7*a*	6007 bc	2535 bc
2019	F0.8	7.15 ± 0.43*a*	145.8 ± 4.2*a*	4.78 ± 0.18*a*	42.5 ± 2.3*a*	6977 a	2955 a
	F1.0	6.59 ± 0.18*b*	139.7 ± 3.2*ab*	4.76 ± 0.15*a*	39.7 ± 1.7*a*	6652 ab	2633 abc
	F1.2	6.47 ± 0.10*b*	138.2 ± 3.5*ab*	4.75 ± 0.07*a*	42.3 ± 0.8*a*	6569 ab	2779 ab
	F1.4	5.64 ± 0.20*c*	118.1 ± 7.7*c*	4.73 ± 0.04*a*	41.0 ± 4.5*a*	5580 c	2294 cd

	CK	5.92 ± 0.20*c*	112.1 ± 4.3*c*	4.89 ± 0.26*a*	45.2 ± 0.6*a*	5480 d	2477 c
	CF	6.05 ± 0.22*c*	114.0 ± 4.2*bc*	4.95 ± 0.09*a*	44.5 ± 0.3*a*	5635 cd	2509 c
	F0.6	6.49 ± 0.25*bc*	120.5 ± 4.1*abc*	5.14 ± 0.68*a*	44.6 ± 0.5*a*	6187 bc	2761 bc
2020	F0.8	7.29 ± 0.49*a*	140.5 ± 13.6*a*	5.12 ± 0.43*a*	45.2 ± 0.3*a*	7142 a	3226 a
	F1.0	7.12 ± 0.27*ab*	133.7 ± 12.2*ab*	5.03 ± 0.37*a*	43.4 ± 1.4*a*	6675 ab	2892 b
	F1.2	6.92 ± 0.20*ab*	133.7 ± 10.3*ab*	4.94 ± 0.16*a*	45.3 ± 3.8*a*	6607 ab	2972 b
	F1.4	6.02 ± 0.09*c*	113.2 ± 7.2*bc*	4.90 ± 0.13*a*	45.0 ± 0.6*a*	5541 cd	2546 cd

*Values followed by different small letters in a column indicate significant difference among treatments (P < 0.05) according to the Duncan’s multiple-range test.*

### Principal Component Analysis

**FIGURE 6:**
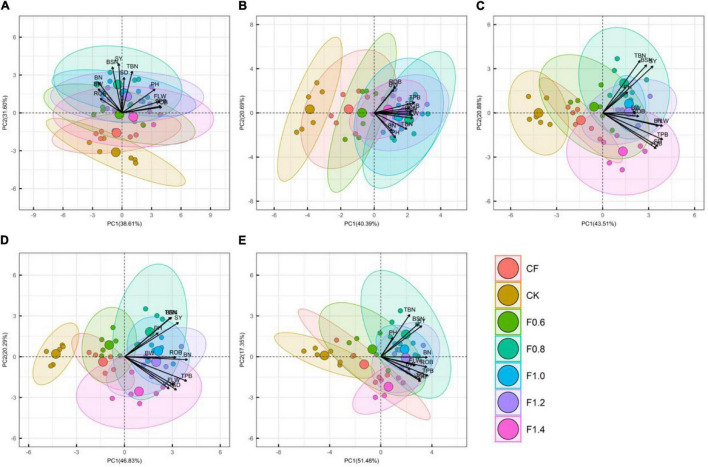
The principal component analysis among measured parameters (i.e., BN, BW, BSN, FLW, PH, ROB, SD, SY, TBN, TPB, and VOB) in different growth stages. The different capital letters in the picture represent different periods of fertility. **(A)** Full squaring stage, **(B)** full flowering stage, **(C)** full boll stage, **(D)** later full boll stage, and **(E)** boll opening stage.

Principal component analysis of different parameters is shown in Figure 6. Biomass accumulation, yield, and its composition, indicates that at full budding stage, the contribution rates of the first, second, and third principal components were 38.6, 31.6, and 12.9%, respectively. The cumulative contribution of the first three principal components was 83.1%, which could explain the data well and clearly distinguish between different fertilization modes in PC2. The maximum VOB in PC1 was 0.95, followed by total biomass, functional leaf width, and plant height, but negatively correlated with the number of fruit branches, weight of single boll, and ROB. The maximum load value of seed cotton yield in PC2 was 0.92, followed by the number of bolls per plant, number of fruit branches, and stem diameter. This showed that the number of fruit branches, number of bolls, and stem diameter could be increased synchronously after applying organic liquid fertilizer at full budding stage and that the smooth flow of pool expansion could help increase the yield.

The contribution rates of the first, second, third, and fourth principal components were 40.4, 20.7, 17.5, and 6.8%, respectively, and the cumulative contribution of the four principal components was 85.4%. Different fertilization modes could be clearly distinguished in PC1. The maximum load value of cotton biomass in PC1 was 0.80, followed by VOB, seed cotton yield, functional leaf width, and the number of bolls per plant. The maximum biomass load of reproductive organs of PC2 was 0.78, followed by single boll weight. The results showed that the application of organic liquid fertilizer could promote the vegetative growth of cotton.

The first, second, third, and fourth principal components contributed 43.5, 20.9, 17.1, and 6.3%, respectively, and the cumulative contribution of the four principal components was 87.7%. The maximum load value of cotton total biomass in PC1 was 0.83, and the load value of VOB was greater than that of seed cotton yield and ROB. PC2 could clearly distinguish F1.4 treatment from other treatments, and the maximum load value of total bolls per unit area in PC2 was 0.75, followed by the number of bolls per plant and seed cotton yield, while stem diameter, VOB, and total biomass were negatively correlated. The contribution rates of the first, second, third, and fourth principal components were 46.8, 20.3, 11.6, and 7.1%, respectively, and the cumulative contribution rate of the four principal components was 85.9%. The maximum load value of the number of fruit branches in PC1 was 0.87, followed by cotton total biomass, seed cotton yield, and cotton ROB. PC2 can also clearly distinguish F1.4 treatment from other treatments, and the load value of the number of bolls per plant and total number of bolls per unit area is larger. However, the yield was negatively correlated with biomass accumulation and agronomic traits. The results indicated that excessive fertilizer input in F1.4 treatment resulted in excessive growth of cotton nutrition, which was not conducive to the increase of yield.

The first, second, third, and fourth principal components contributed 51.5, 17.4, 8.9, and 8%, respectively, and the cumulative contribution rate of the four principal components was 85.7%. The maximum load value of cotton total biomass in PC1 was 0.89, followed by the number of fruit branches, ROB, and seed cotton yield. The maximum load value of total bolls per unit area in PC2 was 0.75, followed by the number of bolls per plant, seed cotton yield, and plant height.

## Discussion

### Organic Liquid Fertilizer Can Promote Cotton Flowering and Maturity and Regulate Cotton Growth

As one of the important agronomic characteristics, cotton growth period is affected by different fertilization treatments (Li, 2013). This study shows that the combined application of organic liquid fertilizer and CF can hasten the flowering and BO stages of cotton. Principal component analysis of the flowering stage also indicates that the combined application of organic liquid fertilizer and CF can promote the vegetative growth of cotton. Organic fertilizer and bioorganic fertilizer instead of CF could prolong the growth of cotton, and the use of more bioorganic fertilizer can result in a longer growth period (Sun et al., 2020). Sufficient nutrient supply in early growth stage can accelerates early development of cotton (Lu et al., 2017); this may be due to nitrogen from moving with water, which resulted in improved capacity of soil fertilizer supply (Li et al., 2019). Therefore, the application of organic fertilizer can enhance soil quality (Han et al., 2021) and improve soil microbial structure (Tao et al., 2020).

The related indexes of cotton plant type directly reflect the growth and development of cotton plants (Feng et al., 2016). This study finds that the combined application of organic and CF s can significantly increase plant height and stem diameter of cotton with the increase application amount, but the yield did not increase synchronously. Compared with F1.0 treatment, the plant height and stem diameter of F0.8 treatment did not decrease significantly. Organic–inorganic compound fertilizer increased soil-plant analysis development (SPAD), plant height, and tiller number of leaves (Moe et al., 2019). Nitrogen application can promote cell division and internode elongation, which may be the main reason for the increase in plant height (Sheoran et al., 2017).

The relationship between sink and source can reflect the coordination between vegetative growth and reproductive growth of cotton and affect the maturity (Chen and Dong, 2016). In this study, organic liquid fertilizer extends the time of cotton leaf width before reaching the peak. These data indicate that organic liquid fertilizer can promote cotton growth, but excessive application can lead to vigorous vegetative growth and serious shedding of lower fruit branches in the later growth stage. The reason may be that excessive application of liquid fertilizer leads to the increase in sink-to-source ratio of cotton, which accelerates leaf senescence, resulting in the increase of leaf shedding in the lower part of cotton (Chen et al., 2018). Another reason might be poor light transmittance in the lower canopy, which reduces the temperature and increases humidity, leading to shedding of buds and bolls (Khan et al., 2017).

### Organic Liquid Fertilizer Prolongs the Rapid Accumulation of Dry Matter and Improves the Accumulation Rate

Dry matter is the basis of cotton yield (Mamnabi et al., 2020), and the rational distribution of photosynthetic compounds is the main factor affecting cotton yield (Zahoor et al., 2017). Replacing CF with organic fertilizer can increase the biomass accumulation of cotton (Wang S. J. et al., 2021). Compared with CF treatment, the total biomass and VOB of cotton under combined application of organic liquid fertilizer and CF showed an upward trend, and F0.8 treatment significantly increased ROB by 20.1%. This indicates that F0.8 treatment (CF reduced by 20% combined with organic liquid fertilizer) improves the transport of photosynthetic products to reproductive organs without reducing the biomass accumulation.

In this study, the combined application of organic liquid fertilizer and CF treatment increased the duration of rapid accumulation of total biomass. The F0.8 treatment had the longest duration and average rate of ROB accumulation. Excessive application of nitrogen fertilizer can hasten rapid growth period and prolong its duration, which affects the full transfer of nutrients to buds and bolls, resulting in reduction of yield (Xue et al., 2006). In this study, principal component analysis shows that there is a significant correlation between cotton biomass accumulation, growth traits, and yield. A positive correlation between cotton yield and the whole plant biomass has been reported (Luo et al., 2020).

### Organic Liquid Fertilizer Can Increase Bolls Number and Yield

Combined application of organic and inorganic fertilizer can promote cotton growth and increase the number of bolls per plant (Tao et al., 2015). This study showed that cotton yield increased with the combined application of organic liquid fertilizer and CF, with the F0.8 treatment having the highest yield. The increase in yield was allied with more bolls per plant and total bolls per unit area. Grain yield increased by 5.58–18.67% after applying urea containing humic acid due to the increase in the number of grains per plant (Zhang S. Q. et al., 2019). Compared with single application of CF, the combination of organic fertilizer with inorganic fertilizer did not promote plant growth but improved the fruit quality (Zhang et al., 2020). However, further increment in the application of organic liquid fertilizer did not increase yield. The might be due to that excessive application of fertilizer can lead to high nutrient concentration in the soil solution, accelerate root senescence, reduce nutrient absorption efficiency, and consequently result in yield loss (Wang H. D. et al., 2021).

## Conclusion

Under film mulching with drip irrigation system, the application of organic liquid fertilizer can accelerate early growth and development of cotton. The F0.8 treatment (CF reduced by 20% combined with organic liquid fertilizer), duration, and rate of rapid accumulation of biomass with increased number of bolls per unit area led to high cotton yield. Further increase in the amount of application rate did not favor cotton yield due to luxury vegetative growth and more boll shedding. Therefore, F0.8 treatment (CF reduced by 20% combined with organic liquid fertilizer, N, P_2_O_5_, and K_2_O were 182, 104, and 76 kg hm^–2^, respectively) is a promising option in terms of improved agronomic traits, biomass accumulation, and seed cotton yield under mulch drip irrigation system.

## Data Availability Statement

The original contributions presented in the study are included in the article/supplementary material, further inquiries can be directed to the corresponding authors.

## Author Contributions

X-JS and X-ZH were jointly responsible for the test design and operation, and complete the writing of the manuscript. N-NL, J-HL, FS, and YT helped complete the test operation and provided suggestions on test design during the test. H-HL, JW, HY-H, and YC provided the overall idea for the experiment. All authors contributed to the article and approved the submitted version.

## Conflict of Interest

The authors declare that the research was conducted in the absence of any commercial or financial relationships that could be construed as a potential conflict of interest.

## Publisher’s Note

All claims expressed in this article are solely those of the authors and do not necessarily represent those of their affiliated organizations, or those of the publisher, the editors and the reviewers. Any product that may be evaluated in this article, or claim that may be made by its manufacturer, is not guaranteed or endorsed by the publisher.
